# Supplementary material for: Soil organic carbon is a key determinant of CH4 sink in global forest soils
Source: Nat Commun. 2023 May 30;14:3110. doi: 10.1038/s41467-023-38905-8 (PMC10229549; doi:10.1038/s41467-023-38905-8)
Supplement: Supplementary file 1 — Supplementary Information [file 41467_2023_38905_MOESM1_ESM.pdf]

Supplementary Information for

Soil organic carbon is a key determinant of CH_4 sink in global forest soils

Jaehyun Lee[1], Youmi Oh[2,3], Sang Tae Lee[4], Yeon Ok Seo[5] Jeongeun Yun[1], Yerang Yang[1], Jinhyun Kim[1,6], Qianlai Zhuang[7], Hojeong Kang[1*]

[1]School of Civil and Environmental Engineering, Yonsei University, Seoul, 03722, Korea

[2]Global Monitoring Laboratory, National Oceanic and Atmospheric Administration, Boulder, CO, USA

[3]Cooperative Institute for Research in Environmental Sciences, University of Colorado, Boulder, CO, USA

[4]Forest Technology and Management Research Center, National Institute of Forest Science, Gyeonggi, Korea

[5]Warm Temperate and Subtropical Forest Research Center, National Institute of Forest Science, Jeju, Korea

[6]Division of Life Sciences, Korea Polar Research Institute, Incheon 21990, Republic of Korea

[7]Department of Earth, Atmospheric, and Planetary Sciences, Purdue University, West Lafayette, IN, USA

Content of the file: Supplementary table 1 to 3, supplementary figure 1 to 9

Supplementary tables

Supplementary Table 1. Partial correlation between SOM content and soil CH_4 uptake rate. Result of partial correlation analysis between soil organic matter (SOM) content and soil CH_4 uptake rate in subtropical forest and temperate forest (AFP: air-filled porosity DOC: dissolved organic carbon, WFPS: water-filled pore space).

Control variables	Study site	Correlation coefficient	*P*-value
None	Subtropical forest	0.757	<0.001
	Temperate forest	0.494	<0.001
AFP, DOC, WFPS	Subtropical forest	0.645	<0.001
	Temperate forest	0.390	<0.001

Supplementary Table 2. Information about observation sites for model optimization.

	**Tropical Forest**	**Temperate Forest**	**Boreal Forest**
**CH_4 data**	Sousa et al.[1]	Borken et al.[2]	Dinsmore et al.[3]
**Climatological input**	Climatic Research Unit V4	observation	observation
**Soil organic carbon (from soilgrid, g kg^{-1})**	36.23	47.86	112.77
**Data period**	2006-2007	2001-2002	2012

Ensemble model optimization results

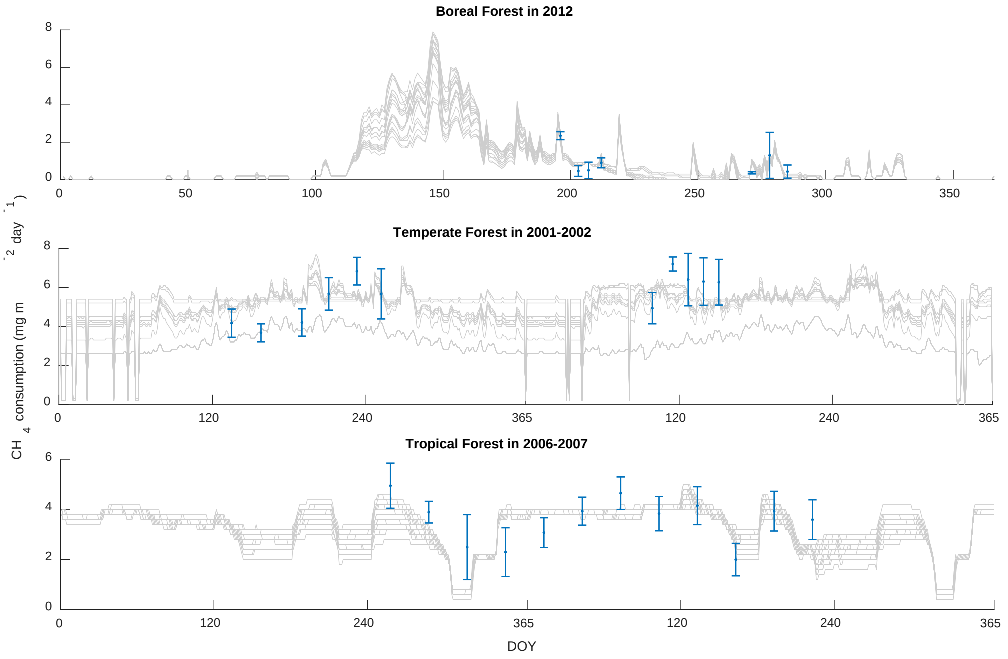

Supplementary Table 3. Optimized parameters for TEM-SOC model. Parameters are (1) maximum potential of methane oxidation by HAM (Omax), (2) Q_{10} temperature sensitivity of methane oxidation (OCH4Q10), (3) Maximum soil moisture for methane oxidation (MVmax), (4) minimum soil moisture for methane oxidation (MVmin), and (5) optimum soil moisture for methane oxidation (MVopt).

**Biome type**	**Omax**	**OCH4Q10**	**MVmax**	**MVmin**	**MVopt**
**Tropical Forest**	2.141±0.514	0.680±0.144	0.781±0.118	0.077±0.024	0.308±0.064
**Temperate Forest**	7.301±0.736	1.154±0.172	0.765±0.125	0.028±0.024	0.339±0.068
**Boreal Forest**	3.555±1.170	3.217±0.865	0.773±0.099	0.218±0.009	0.487±0.034

Supplementary figures

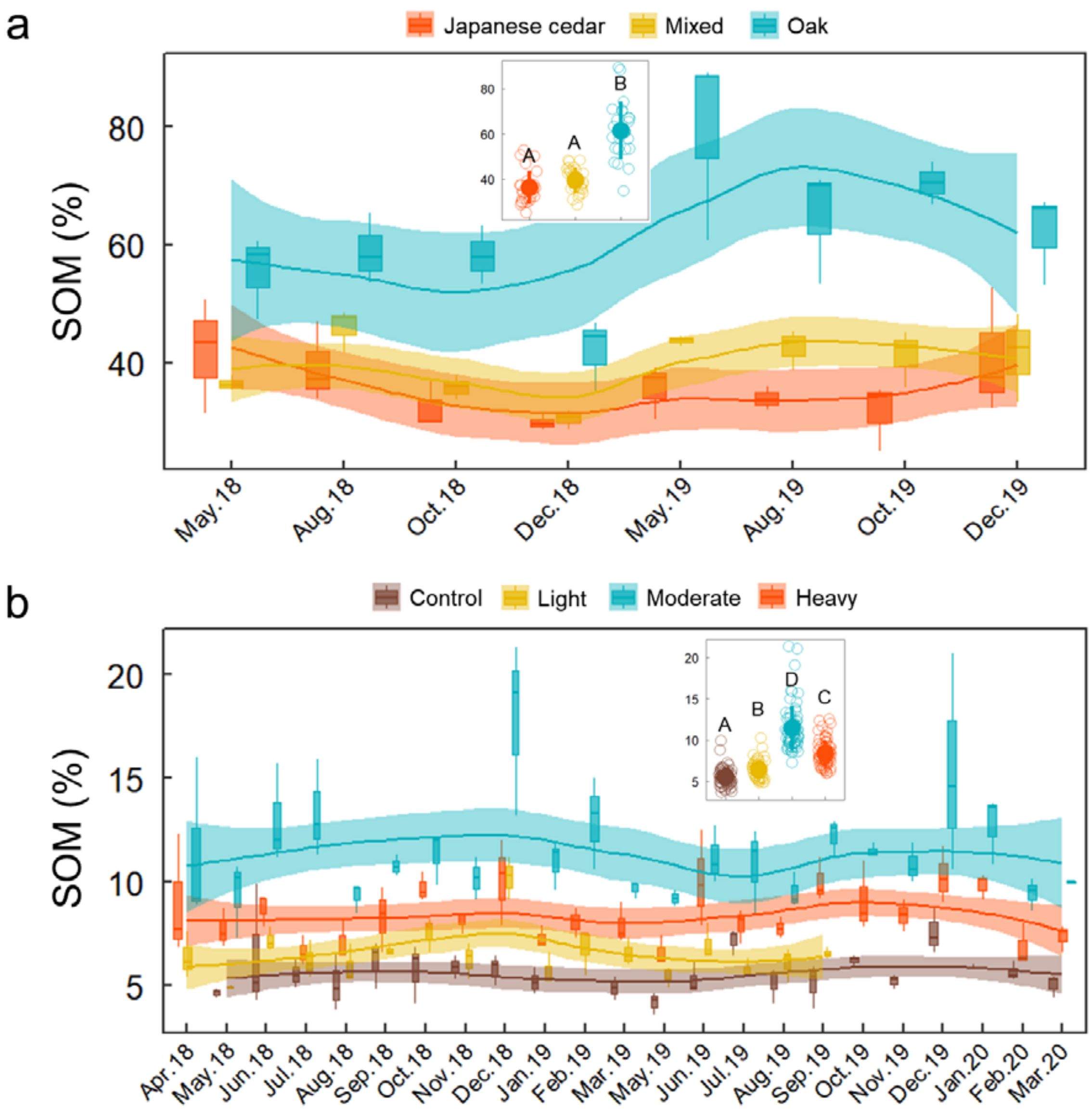

Supplementary Fig. 1. Soil organic matter content observed in subtropical forest and temperate forest. (a) Seasonal variation of soil organic matter (SOM) content in the subtropical forest with different tree species and (b) the temperate forest with different thinning intensities. The thick central line represents the median value, the boxed areas represent the interquartile range, and the whiskers show the maximum and minimum values (N = 3). The lines indicate LOESS locally weighted polynomial regression curves and error bands surrounding the regression lines represent the 95% confidence interval of the correlation. Dot plots present the average SOM content at the different tree species and thinning intensities. Error bars are standard errors of the mean. Alphabets denote statistically significant differences ($P < 0.05$) between the tree species ($N = 24$) or thinning intensity ($N = 72$ for control, moderate, and heavy intensity, and $N = 54$ for light intensity) based on Tukey's HSD test.

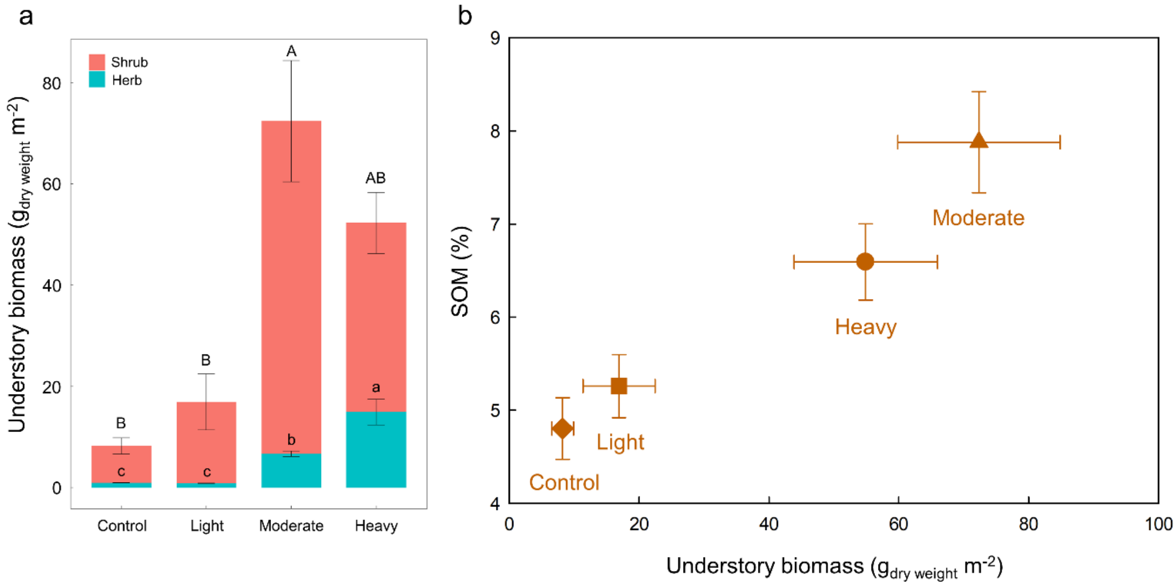

Supplementary Fig. 2. Understory biomass with different thinning intensities. (a) Understory (shrub and herb) biomass in temperate forests with different thinning intensities and (b) the relationship between soil organic matter (SOM) content and understory biomass. Alphabets denote statistically significant differences between thinning intensities (P < 0.05) based on Tukey's HSD test and the error bars indicate standard error of mean (N = 3).

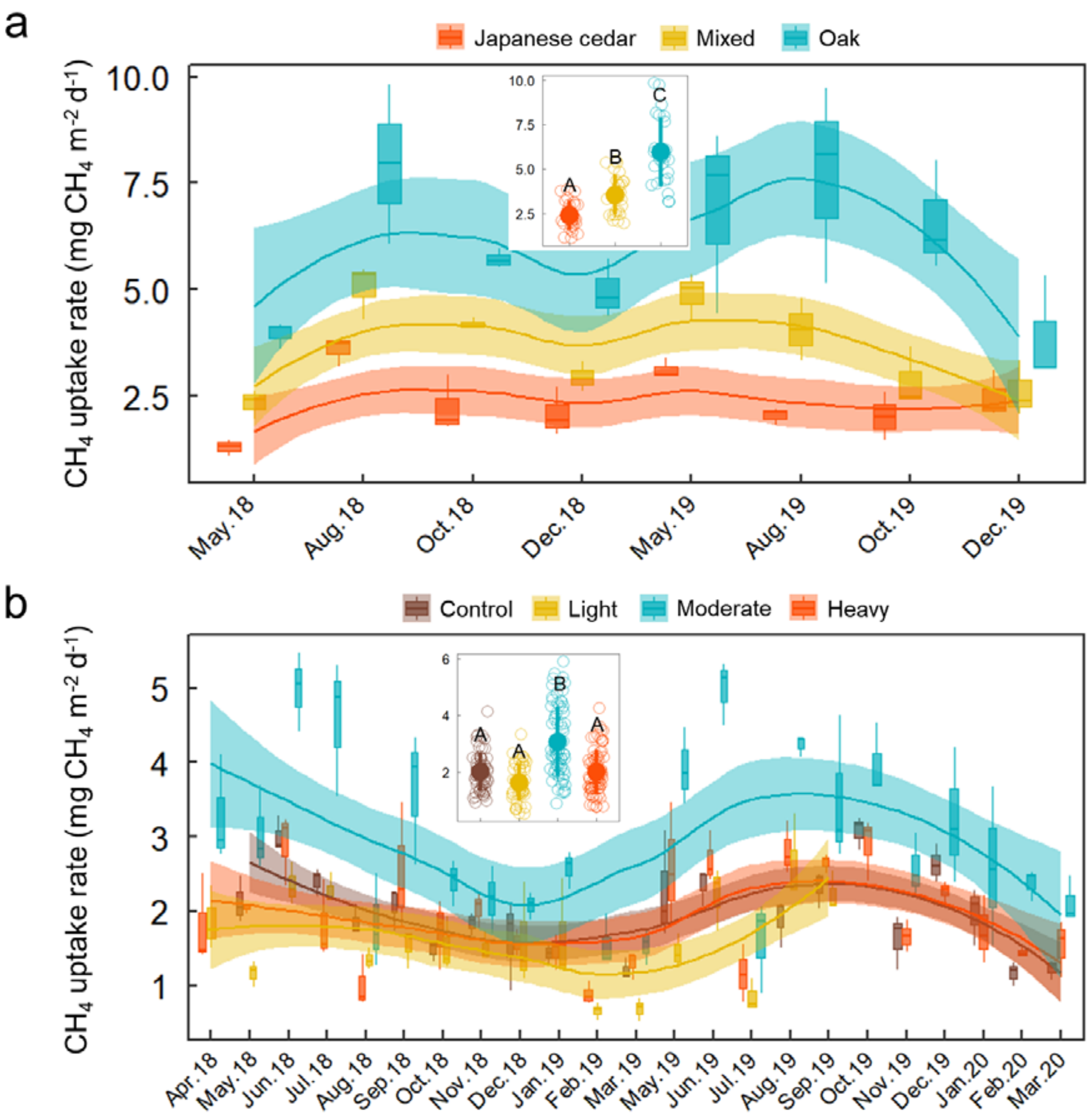

Supplementary Fig. 3. Soil CH_4 uptake rate observed in subtropical forest and temperate forest. Seasonal variation of soil CH_4 uptake rate in (a) the subtropical forest with different tree species and (b) the temperate forest with different thinning intensities. The thick central line represents the median value, the boxed areas represent the interquartile range, and the whiskers show the maximum and minimum values ($N = 3$). The lines indicate LOESS locally weighted polynomial regression curves and error bands surrounding the regression lines represent the 95% confidence interval of the correlation. Dot plots present the average soil CH_4 uptake rate at the different tree species and thinning intensities. Error bars are standard error of mean. Alphabets denote statistically significant differences ($P < 0.05$) between the tree species ($N = 24$) or thinning intensity ($N = 72$ for control, moderate, and heavy intensity, and $N = 54$ for light intensity) based on Tukey's HSD test.

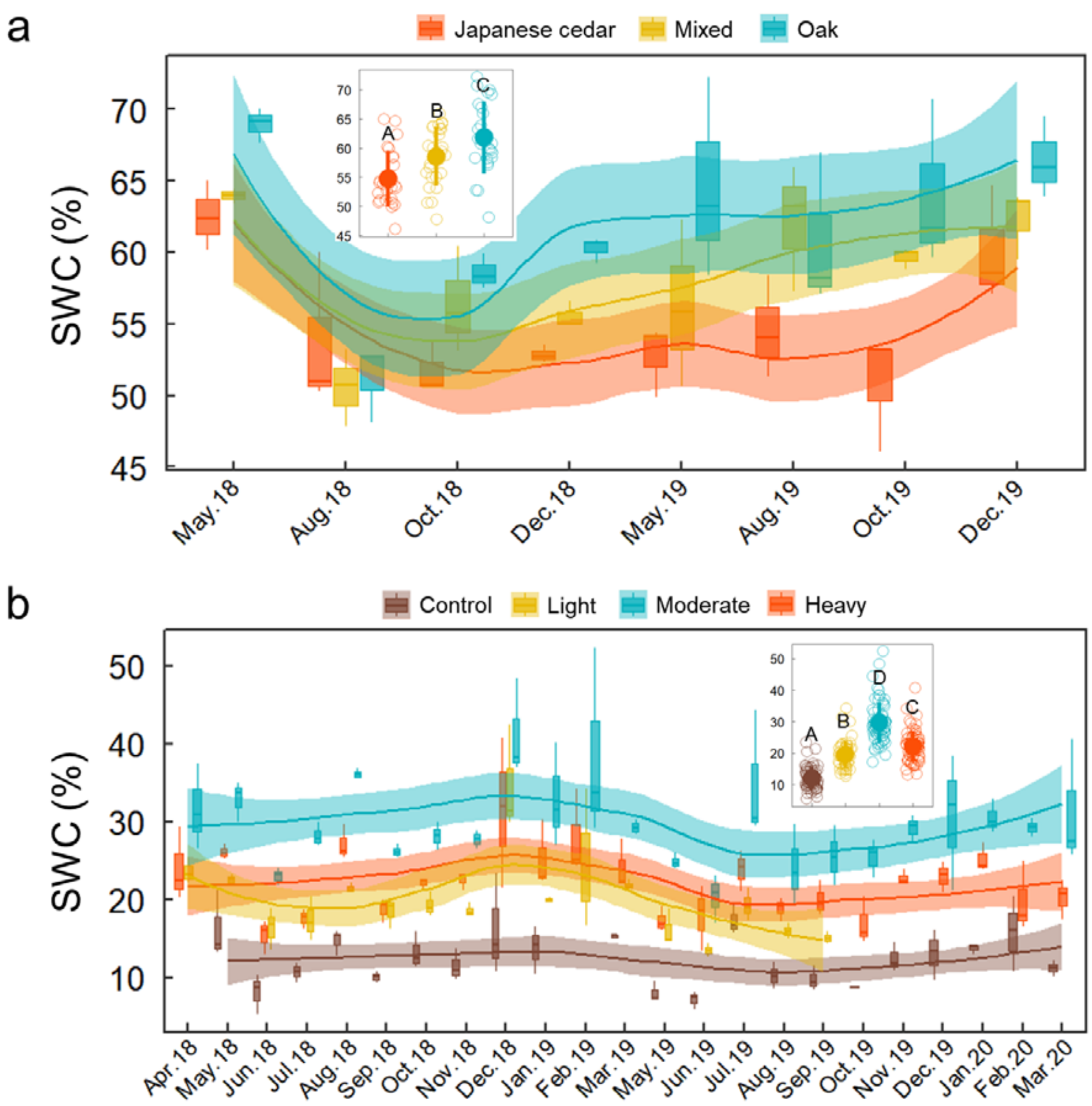

Supplementary Fig. 4. Soil water content observed in subtropical forest and temperate forest. Seasonal variation of soil water content (SWC) in (a) the subtropical forest with different tree species and (b) the temperate forest with different thinning intensities. The thick central line represents the median value, the boxed areas represent the interquartile range, and the whiskers show the maximum and minimum values (N = 3). The lines indicate LOESS locally weighted polynomial regression curves and error bands surrounding the regression lines represent the 95% confidence interval of the correlation. Dot plots present the average SWC at the different tree species and thinning intensities. Error bars are standard error of mean. Alphabets denote statistically significant differences ($P < 0.05$) between the tree species (N = 24) or thinning intensity (N = 72 for control, moderate, and heavy intensity, and N = 54 for light intensity) based on Tukey's HSD test.

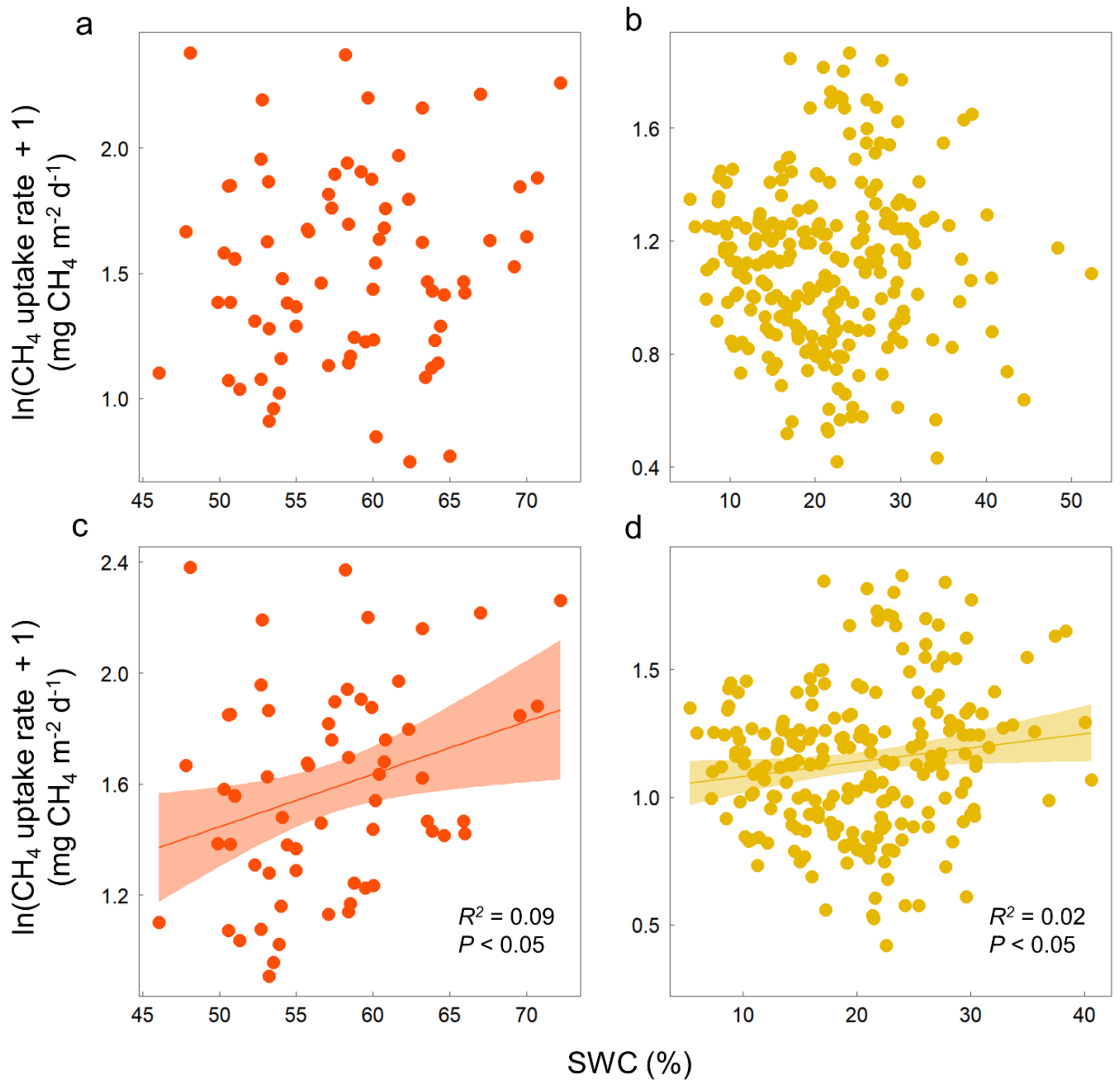

Supplementary Fig. 5. Relationship between soil CH_4 uptake rate and soil water content. Correlation between soil CH_4 uptake rate and soil water content (SWC) in (a) subtropical forest with different tree species (N = 72) and (b) temperate forest with different thinning intensities (N = 258). The lower panel presents the relationship between soil CH_4 uptake rate and SWC when the highly saturated periods are excluded (April 2018 in subtropical forest and December 2018, February 2019 in temperate forest) in (c) subtropical forest (N = 69, $P < 0.05$) and (d) temperate forest (N = 252, $P < 0.05$), respectively. A linear regression was used and the error bands surrounding the regression lines represent the 95% confidence interval of the correlation.

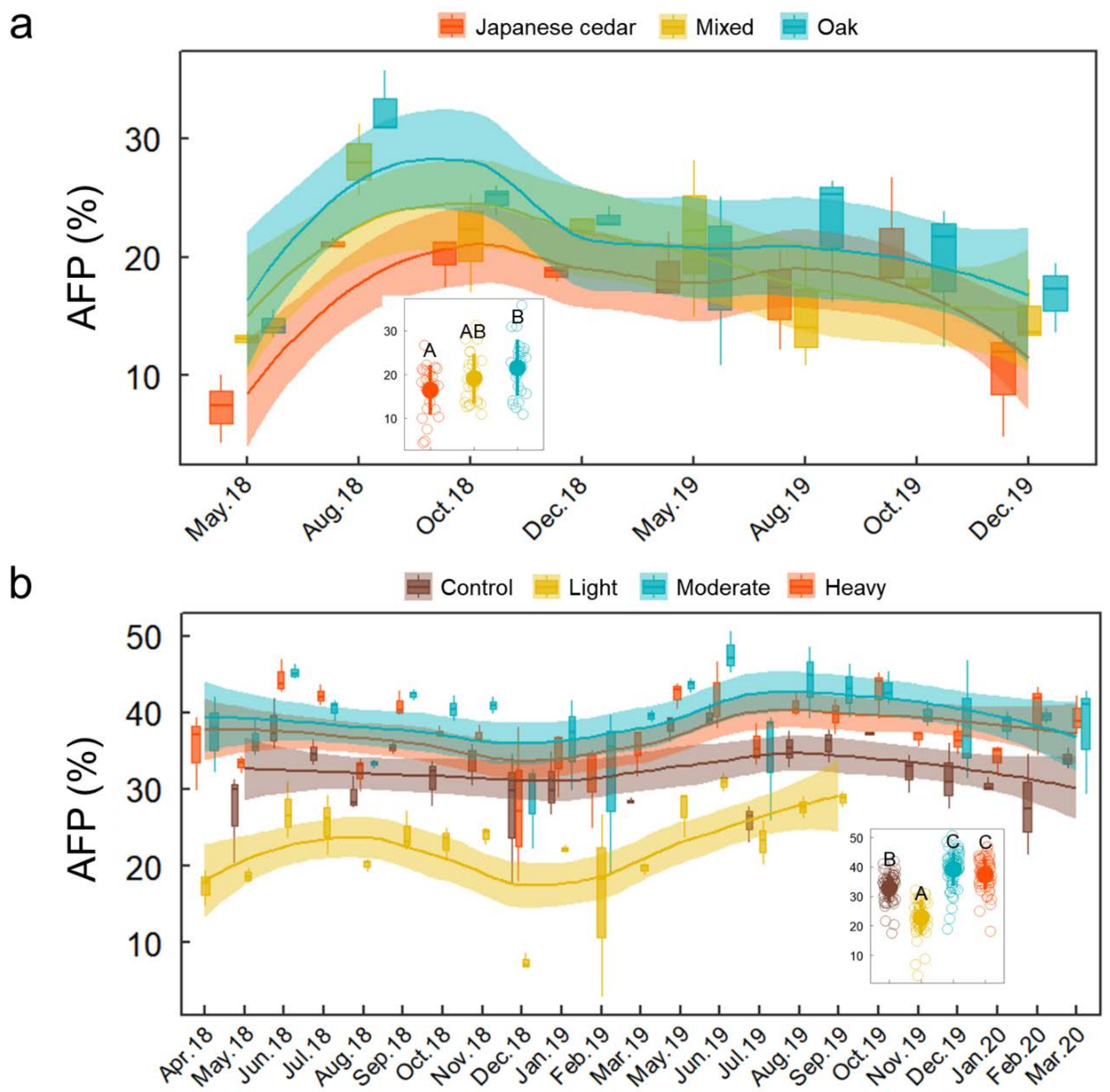

Supplementary Fig. 6. Soil air-filled porosity observed in subtropical forest and temperate forest. Seasonal variation of soil air-filled porosity (AFP) in (a) the subtropical forest with different tree species and (b) the temperate forest with different thinning intensities. The thick central line represents the median value, the boxed areas represent the interquartile range, and the whiskers show the maximum and minimum values ($N = 3$). The lines indicate LOESS locally weighted polynomial regression curves and error bands surrounding the regression lines represent the 95% confidence interval of the correlation. Dot plots present the average soil AFP at the different tree species and thinning intensities. Error bars are standard error of mean. Alphabets denote statistically significant differences ($P < 0.05$) between the tree species ($N = 24$) or thinning intensity ($N = 72$ for control, moderate, and heavy intensity, and $N = 54$ for light intensity) based on Tukey's HSD test.

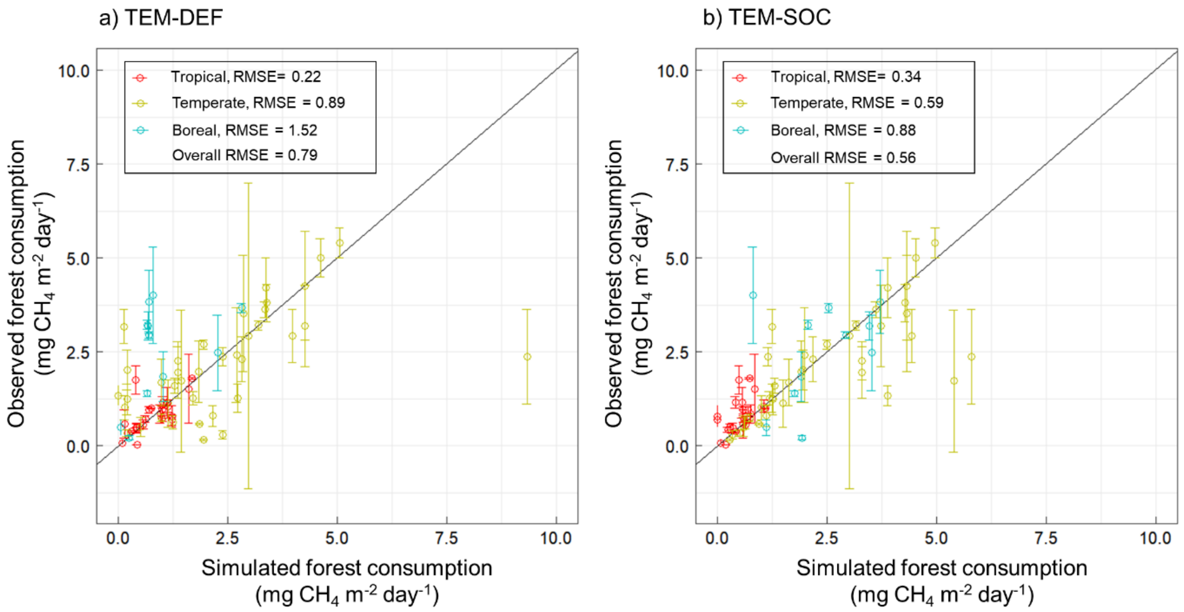

Supplementary Fig. 7. Model-data comparison of CH_4 uptake using site-level data. Comparison of observed forest CH_4 uptake and simulated forest CH_4 uptake result from (a) TEM-DEF and (b) TEM-SOC with different biome types (N = 71). Error bars represent standard deviation of measured forest CH_4 consumption rates from the literature.

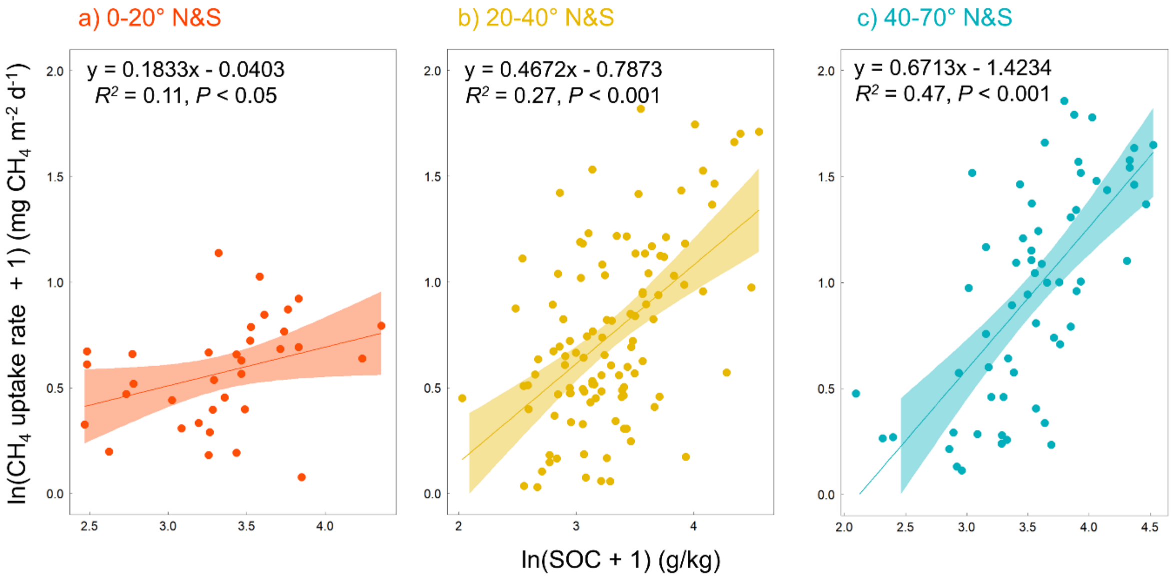

Supplementary Fig. 8. Relationship between soil CH_4 uptake rate and soil organic carbon content in different latitudinal ranges. Relationship between forest soil CH_4 uptake rate and soil organic carbon (SOC) content at (a) 0-20° N&S (N = 35, P < 0.05), (b) 20-40° N&S (N = 109, P < 0.001), and (c) 40-70° N&S (N = 60, P < 0.001). A linear regression was used and the error bands surrounding the regression lines represent the 95% confidence interval of the correlation.

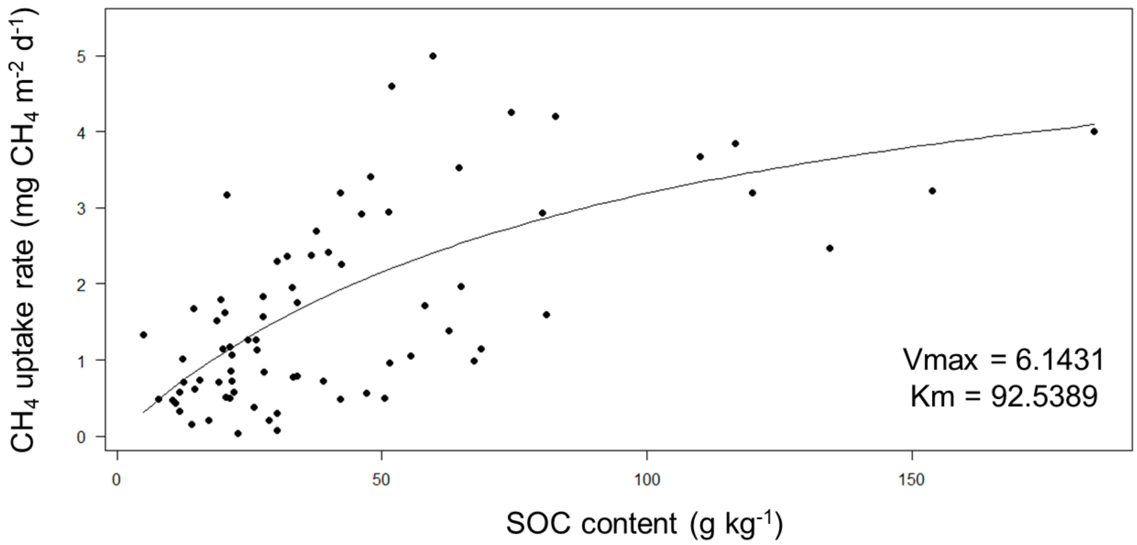

Supplementary Fig. 9. Michaelis-Menten function between soil organic carbon content and CH_4 uptake rate. Michaelis-Menten curve between soil CH_4 uptake rate and soil organic carbon (SOC) content derived from global metadata.

Supplementary References

1. Sousa Neto, E. *et al.* Soil-atmosphere exchange of nitrous oxide, methane and carbon dioxide in a gradient of elevation in the coastal Brazilian Atlantic forest. *Biogeosciences* **8**, 733–742 (2011).
2. Borken, W. & Beese, F. Methane and nitrous oxide fluxes of soils in pure and mixed stands of European beech and Norway spruce. *Eur. J. Soil Sci.* **57**, 617–625 (2006).
3. Dinsmore, K. J. *et al.* Growing season CH4 and N2O fluxes from a subarctic landscape in northern Finland; From chamber to landscape scale. *Biogeosciences* **14**, 799–815 (2017).
