# Supplementary material for: Soil organic carbon is a key determinant of CH4 sink in global forest soils
Source: Nat Commun. 2023 May 30;14:3110. doi: 10.1038/s41467-023-38905-8 (PMC10229549; doi:10.1038/s41467-023-38905-8)
Supplement: Supplementary file 3 — Description of Additional Supplementary Files [file 41467_2023_38905_MOESM3_ESM.pdf]

Description of Additional Supplementary Files:

Supplementary Data 1: Meta-analysis dataset. A dataset used for meta-analysis for the elucidation of the relationship between soil organic carbon content and soil CH_4 sink across the global forests.
