# Supplementary material for: Soil organic carbon is a key determinant of CH4 sink in global forest soils
Source: Nat Commun. 2023 May 30;14:3110. doi: 10.1038/s41467-023-38905-8 (PMC10229549; doi:10.1038/s41467-023-38905-8)
Supplement: Supplementary file 5 — Reporting Summary [file 41467_2023_38905_MOESM5_ESM.pdf]

nature portfolio

Corresponding author(s): Hojeong Kang

Last updated by author(s): May 4, 2023

Reporting Summary

Nature Portfolio wishes to improve the reproducibility of the work that we publish. This form provides structure for consistency and transparency in reporting. For further information on Nature Portfolio policies, see our Editorial Policies and the Editorial Policy Checklist.

Statistics

For all statistical analyses, confirm that the following items are present in the figure legend, table legend, main text, or Methods section.

n/a	Confirmed	
☐	☒	The exact sample size (*n*) for each experimental group/condition, given as a discrete number and unit of measurement
☐	☒	A statement on whether measurements were taken from distinct samples or whether the same sample was measured repeatedly
☐	☒	The statistical test(s) used AND whether they are one- or two-sided<br>*Only common tests should be described solely by name; describe more complex techniques in the Methods section.*
☐	☒	A description of all covariates tested
☐	☒	A description of any assumptions or corrections, such as tests of normality and adjustment for multiple comparisons
☐	☒	A full description of the statistical parameters including central tendency (e.g. means) or other basic estimates (e.g. regression coefficient) AND variation (e.g. standard deviation) or associated estimates of uncertainty (e.g. confidence intervals)
☐	☒	For null hypothesis testing, the test statistic (e.g. *F*, *t*, *r*) with confidence intervals, effect sizes, degrees of freedom and *P* value noted<br>*Give P values as exact values whenever suitable.*
☒	☐	For Bayesian analysis, information on the choice of priors and Markov chain Monte Carlo settings
☐	☒	For hierarchical and complex designs, identification of the appropriate level for tests and full reporting of outcomes
☐	☒	Estimates of effect sizes (e.g. Cohen's *d*, Pearson's *r*), indicating how they were calculated

Our web collection on statistics for biologists contains articles on many of the points above.

Software and code

Policy information about availability of computer code

Data collection	Supporting Information is available online. The dataset used for meta-analysis can be found in Supplementary Data. The model results are archived and freely available at the Purdue University Research Repository (PURR) at: https://purr.purdue.edu/publications/4252/1 (https://doi.org/10.4231/8K7W-NF84).
Data analysis	The code is written in C++ language. Code guideline is available at the Purdue University Research Repository (PURR) at: https://purr.purdue.edu/publications/4252/1 (https://doi.org/10.4231/8K7W-NF8461).

For manuscripts utilizing custom algorithms or software that are central to the research but not yet described in published literature, software must be made available to editors and reviewers. We strongly encourage code deposition in a community repository (e.g. GitHub). See the Nature Portfolio guidelines for submitting code & software for further information.

Data

Policy information about availability of data

All manuscripts must include a data availability statement. This statement should provide the following information, where applicable:

- Accession codes, unique identifiers, or web links for publicly available datasets
- A description of any restrictions on data availability
- For clinical datasets or third party data, please ensure that the statement adheres to our policy

Supporting Information is available online. The dataset used for meta-analysis can be found in Supplementary Data. The model results are archived and freely available at the Purdue University Research Repository (PURR) at: https://purr.purdue.edu/publications/4252/1 (https://doi.org/10.4231/8K7W-NF84).

Human research participants

Policy information about studies involving human research participants and Sex and Gender in Research.

Reporting on sex and gender	N/A
Population characteristics	N/A
Recruitment	N/A
Ethics oversight	N/A

Note that full information on the approval of the study protocol must also be provided in the manuscript.

Field-specific reporting

Please select the one below that is the best fit for your research. If you are not sure, read the appropriate sections before making your selection.

☐ Life sciences ☐ Behavioural & social sciences ☒ Ecological, evolutionary & environmental sciences

For a reference copy of the document with all sections, see nature.com/documents/nr-reporting-summary-flat.pdf

Ecological, evolutionary & environmental sciences study design

All studies must disclose on these points even when the disclosure is negative.

Study description	This study is based on 3 different approaches, in-situ observations, global meta-analysis, and process-based modeling. Field observation was performed in two sites, one with different tree species, and the other was with different thinning intensity. Meta-analysis was performed by collecting 81 previously published papers. Lastly, we revised process-based model by considering new parameter. Using three different approaches, we aim to investigate the effect of soil organic carbon on forest CH4 sink capacity.
Research sample	Soil samples were collected to the depth of 0-10 cm after litter layer and O-horizon removal. Soil temperature was measured at the depth of 5 cm after soil sampling. Fluxes were measured using the closed static chamber method with triplicate measurement in each treatment. Changes in headspace CH4 concentration were measured using GasScouterTM G4301 Mobile Gas Concentration Analyzer (Picarro Inc., Santa Clara, CA, USA). Due to mechanical issues with the portable analyzer, we took the headspace gas sample every 10 minutes for 50 minutes and transferred it to a pre-evacuated glass vial.
Sampling strategy	In each plot, static chambers were installed between trees and the distance between chambers was at least 10m. Surface soil samples were collected after removal of O-horizon.
Data collection	Soil chemical and physical characteristics were analyzed as described in Method section. Gas data were stored in portable gas analyzer as text file and converted to flux data (mg CH4 m-2 day-1). CH4 fluxes were calculated from linear regression slopes (chamber headspace [CH4] vs. time) with a minimum R2 = 0.82.
Timing and spatial scale	Sampling was conducted every 3 months in subtropical forest and every month in temperate forest from April 2018 to March 2020.
Data exclusions	There was no data exclusion.
Reproducibility	We included descriptions of standard sampling strategy as well as analytical protocols. We provide dataset for meta-analysis as Supplementary data.
Randomization	N/A
Blinding	N/A

Did the study involve field work? ☒ Yes ☐ No

Field work, collection and transport

Field conditions	Experiments were performed in managed forests in South Korea. The mean annual temperature was 17.0°C and 11.7°C in subtropical forest and temperate forest, respectively, and the mean annual precipitation was 2188 mm and 1364 mm during the observation period in subtropical forest and temperate forest, respectively.
Location	The study sites were located in the Hannam experimental forest (subtropical forest; 33° 33'N, 126°65'E) and in Gwangneung experimental forest (temperate forest; 37°76' E, 127°17'E) in the Republic of Korea.
Access & import/export	We've got permission to set up the observation system from National Forestry Institution.
Disturbance	The study did not cause any environmental disturbance.

Reporting for specific materials, systems and methods

We require information from authors about some types of materials, experimental systems and methods used in many studies. Here, indicate whether each material, system or method listed is relevant to your study. If you are not sure if a list item applies to your research, read the appropriate section before selecting a response.

Materials & experimental systems

n/a	Involved in the study	
☒	☐	Antibodies
☒	☐	Eukaryotic cell lines
☒	☐	Palaeontology and archaeology
☒	☐	Animals and other organisms
☒	☐	Clinical data
☒	☐	Dual use research of concern

Methods

n/a	Involved in the study	
☒	☐	ChIP-seq
☒	☐	Flow cytometry
☒	☐	MRI-based neuroimaging
